# A new set of primers for COI amplification from purpleback flying squid (*Sthenoteuthis oualaniensis*)

**DOI:** 10.1080/23802359.2017.1357439

**Published:** 2017-07-25

**Authors:** Lei Xu, Qi Huang, Shaolin Xu, Xuehui Wang, Peng Zhang, Liang Xu, Feiyan Du

**Affiliations:** a Guangdong Provincial Key Laboratory of Fishery Ecology and Environment, Key Laboratory of South China Sea Fishery Resources Development and Utilization, Ministry of Agriculture, South China Sea Fisheries Research Institute, Chinese Academy of Fishery Sciences, Guangzhou, China;; b Department of Ecology, Jinan University, Guangzhou, China;; c Department of Biology, Shantou University, Shantou, China

**Keywords:** COI, new primer set, *Sthenoteuthis oualaniensis*, DNA barcodes

## Abstract

Despite the contribution of DNA barcoding towards understanding the biodiversity and distribution of species, the success of the mitochondrial cytochrome c oxidase subunit I gene (COI) amplification has been quite variable when it comes to Cephalopoda. Some species in this class such as *Sthenoteuthis oualaniensis* seem to be more difficult to amplify COI than others due to failed amplifications with universal primer and lack of specific set of primers. In this study, we developed new *Sthenoteuthis* – specific primer set, which significantly increased average amplification success. The new primer set will aid the recovery of barcodes from this difficult group and facilitate further studies in phylogeny and cryptic diversity of *Sthenoteuthis oualaniensis*.

## Introduction

The purpleback flying squid (*Sthenoteuthis oualaniensis*; Lesson, 1830) is widely distributed in the equatorial and subtropical areas of the Pacific and the Indian Oceans (Voss 1973), it does not extend into the temperate Pacific, and however, it is most abundant in the South China Sea and north-western Indian Ocean (Nesis 1977; Mohamed et al. 2006). This squid is a species of growing commercial interest (Zuyev et al. 2002; Chen et al. 2007) and of special interest from the viewpoint of effective and rational use of the world ocean’s biological resources (Trotsenko and Pinchukov 1994). Voss (1973) speculated a potential of the purpleback flying squid of at least 100,000 metric tons in the Central Eastern Pacific. It is on record that the purpleback flying squid are caught commercially in the eastern and southern East China Sea, Taiwan to Okinawa by hook and line with light at night (Tung 1981; Okutani and Tung 1987; Chen et al. 2007; Zhang et al. 2010).

*Sthenoteuthis oualaniensis* comprises multiple forms (Nesis 1993; Dunning 1998), varying both in size at maturity and in the possession of a distinctive large dorsal photophore. A middle-sized ‘typical’ form with the photophore is found throughout the species range in the eastern tropical Pacific. Equatorial waters of the Indian and Pacific Oceans are inhabited by an ‘early-maturing’ dwarf form that lacks the dorsal photophore and may constitute a separate, as yet undescribed, species (Nesis 1993; Staaf et al. 2010). DNA barcoding from the two morphological groups of *S. oualaniensis* may provide essential information for the better understanding the diversity and sustainable utilization of this species. However, few success of cytochrome c oxidase I (COI) amplification has been currently available in this species of the two morphological groups (Staaf et al. 2010). One possible explanation is that a specific set of primers do not exist. Currently, COI amplification in cephalopods has been performed using ‘Folmer’ or ‘Folmer-tailed’ primers (Folmer et al. 1994). Some species like *S. oualaniensis* seem to be more difficult to amplify than others using universal primer. The aim of this work was to develop a new and specific primer set that would reliably amplify COI from the two morphological groups of *S. oualaniensis*. This *Sthenoteuthis* – specific primer set will greatly aid the recovery of barcodes from this difficult group and facilitate further studies in global haplotype diversity of *S. oualaniensis*.

## Materials and methods

### Primer design

The complete mitochondrial genome sequences from three individuals of *S. oualaniensis* were downloaded from GenBank database (accession nos. EU658923, EU660576 and EU660577), and the COI genes were excised and aligned with other 70 COI sequences *S. oualaniensis* (also downloaded from GenBank database, accession nos. KR780710–KR780746, EU310878–EU310887 and DQ885824–DQ885847) using BioEdit (Hall 1999). Potential primer regions were analysed in using Primer Premier version 6.0 (Premier Biosoft Inst., Palo Alto, CA) with default parameters. New primer set were then manually designed based on flanking the more polymorphic region of COI gene.

### DNA extraction, PCR and sequencing

Whole genomic DNA was extracted from muscle tissue of 40 specimens of *S. oualaniensis* collected from the South China Sea (11°30′N, 115°59′E) using TIANamp Marine Animals DNA Kit (TIANGEN, Beijing, China). The concentration for use as a PCR template was adjusted to an A_260_ of about 0.05–0.2. All collected specimens and extracted DNA were stored in Guangdong Provincial Key Laboratory of Fishery Ecology and Environment.

To evaluate the effectiveness of primer set, both two morphologically distinct forms (phenogroups) of *S. oualaniensis* were analysed: the ‘medium form’ (middle-sized, with photophore) and the ‘dwarf form’ (small-sized, without photophore). Twenty specimens from each morphological form of *S. oualaniensis* were performed using PCR amplification. These 40 specimens were assembled along with one negative control to create a 96-well test plate. Three PCRs were conducted on this plate. The first reaction employed universal primer set for the mitochondrial 16S rRNA gene (16Sar and 16Sbr; Palumbi 1996) to provide a positive control for DNA extraction. The other two reactions targeted the COI barcode region using traditional Folmer primers (LCO1490/HCO2198) and the primer set designed in this study, respectively (Folmer et al. 1994). PCR amplification was carried out in 50 μl PCR consisted of 31.25 μl dd H_2_O, 5 μl PCR buffer, 5 μl CoralLoad concentrate, 4 μl of 25 μM MgCl_2_, 1 μl of 10 μM dNTPs, 0.5 μl of 25 μM solution of each primer, 2.5 μl DNA template and 0.25 μl TopTaq DNA polymerase (Qiagen, Hilden, Germany). The PCR conditions for amplification were as follows: initial denaturation 5 min at 95 °C, 40 cycles of 30 s at 95 °C (denaturation), 30 s at 46–50 °C (annealing) and 60 s at 72 °C (extension), followed by 7 min at 72 °C (final extension) on a 2720 Thermal Cycler (Applied Biosystems, Foster City, CA).

To confirm amplification, PCR products were visualized on a 2% agarose gel using Gel Doc™ XR + (Bio-Rad, Hercules, CA), whereas positive PCR products were cycle sequenced using a modified (Hajibabaei et al. 2005) BigDye^©^ Terminator v.3.1 Cycle Sequencing Kit (Applied Biosystems, Inc.) and sequenced bi-directionally on an ABI 3730XL automated sequencer with both forward and reverse of *Sthenoteuthis* – specific primer.

## Results

**Figure 1.**
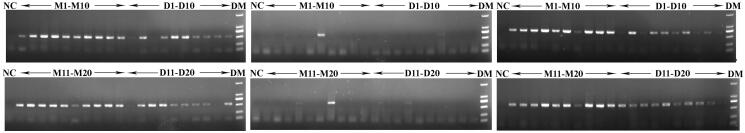
Images of PCR amplicons for representatives of 40 *S. oualaniensis* specimens. (Left column: PCR amplicons of 16S positive control for DNA extraction; middle column: PCR amplicons of COI with Folmer primers; right column: PCR amplicons of COI with *Sthenoteuthis*-specific primer set; NC: negative control; M: medium form; D: dwarf form; DM: DL2000 DNA Marker).

The forward SthenoF (5′-CCATAAAGACATTGGTACTC-3′) and reverse SthenoR (5′-ATAAACTTCTGGGTGACC-3′) were developed along the most conservative sites of the flanking region of COI gene based on complete mitochondrial genome sequences of *S. oualaniensis*. High intensity bands were generated with the 16S primer set from 36 specimen templates, while DNA from four ‘dwarf form’ specimens failed to amplify 16S, but were weakly amplified by our new COI primer set (Figure 1).

**Figure 2.**
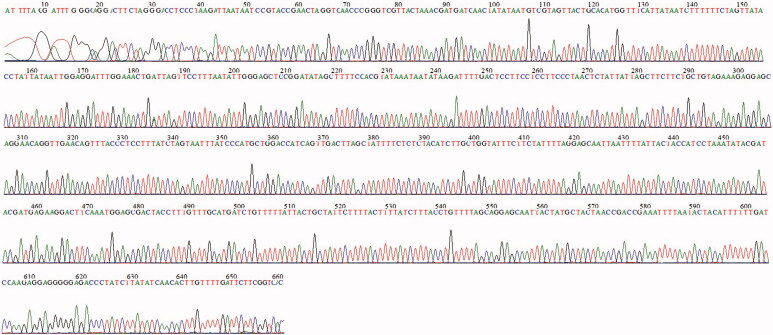
Sequencing peaks of COI gene amplified by the new primer set from studied *S. oualaniensis* sample (sequenced with SthenoF).

To compare the efficacy of our new primer set to traditional Folmer primers, we extracted DNA from 40 specimens of two morphologically distinct forms of *S. oualaniensis*. PCR success rates (12.5%) for traditional Folmer primers are rather low, and only 3 out of 20 samples from ‘medium form’ and 2 out of 20 samples from ‘dwarf form’ of *S. oualaniensis* generated weakly positive PCR products (Figure 1). Nevertheless, our new *Sthenoteuthis* – specific primer set was very effective (100%) in amplifying the target region of COI (Figure 1) and no case of nonspecific amplification was detected. Forty samples from two morphologically distinct forms (phenogroups) of *S. oualaniensis* generated positive PCR products, and even four ‘dwarf form’ specimens failed to amplify 16S, but were weakly amplified by the new primer set. The sequencing success of amplicons was also high for the new *Sthenoteuthis* – specific primer set. Average 688 bp read length and high quality of COI sequence were obtained after sequenced with SthenoF and SthenoR (Figure 2). All the sequences in this research were uploaded to GenBank database (GenBank accession nos. MF402014–MF403049 and MF411091–MF411130)

## Discussion

In recent years, marine fishery resource exploitation has shifted to *S. oualaniensis* because it is characterized by a wide ecological amplitude, complex intraspecific structure, high fecundity, short life cycle, high natural mortality, high growth rate and significant production (Nesis 1977; Chen 2007). Nesis (1993) described multiple forms for *S. oualaniensis* that comprises middle-sized form with the photophore and ‘early-maturing’ dwarf form that lacks the dorsal photophore. However, whether or not the dwarf form is a distinct species is still arguing (Clarke 1965; Nesis 1993; Wormuth 1998). DNA barcoding information and phylogenetic analysis may reveal distinct genetic differentiation between the ‘medium form’ and the ‘dwarf form’ of *S. oualaniensis*. Unfortunately, until Staaf et al. (2010) report population genetic study of *S. oualaniensis* using the mitochondrial marker NADH dehydrogenase subunit 2, little is known about the genetic basis of population structure in the two morphologically distinct forms (phenogroups) of *S. oualaniensis*, although the presence of two morphological species within *S. oualaniensis* has been suggested (Clarke 1965; Wormuth 1976; Nesis 1993). Staaf’s phylogenetic analysis supports *S. oualaniensis* contains multiple deeply divergent, geographically segregated clades, despite the high potential for dispersal in this active swimmer

Theoretically, the 13 protein coding genes in the animal mitochondrial genome are better targets because indels are rare since most lead to a shift in the reading frame, but the cytochrome c oxidase I gene (COI) does have an important advantages (Hebert et al. 2003). COI appears to possess a greater range of phylogenetic signal than any other mitochondrial gene. In common with other protein coding genes, its third-position nucleotides show a high incidence of base substitutions, leading to a rate of molecular evolution that is about three times greater than that of 12S or 16S rDNA (Knowlton and Weigt 1998). In addition, COI-based identification system for members of the animal phyla is generally accepted, and more than 5,000,000 barcode COI sequences have been assembled in BOLD (The Barcode of Life Data System) which provides an integrated bioinformatics platform that supports all phases of the analytical pathway from specimen collection to tightly validate barcode library (Hebert et al. 2003; Ratnasingham and Hebert 2007). Despite DNA barcoding provides digitalized criteria and effective means for species identification, and is becoming an important technical tool in the research on taxonomy and biodiversity, the success of COI amplification has been quite variable when it comes to marine animals; even more, some species in Cephalopoda such as *Eucleoteuthis luminosa*, *Hyaloteuthis pelagica* and *S. oualaniensis* failed to amplify COI gene with traditional Folmer primers (Wakabayashi et al. 2006, 2012; Dai et al. 2012).

Although *S. oualaniensis* is widely distributed in the equatorial and subtropical areas of the Pacific and the Indian Oceans, large-scale COI-based population haplotype diversity studies in the multiple forms species are still lacking, largely due to poor COI amplification success. Our new *Sthenoteuthis* – specific primer set significantly improved the overall results compared with traditional Folmer primers. Amplification rate with traditional Folmer primers from 12.5% was significantly increased to 100% with new *Sthenoteuthis* – specific primer set (Figure 1), and strongly indicated variations do exist in binding position of Folmer primers. Our new *Sthenoteuthis* – specific primer set directly designed based on the flanking region of COI gene of mitochondrial genome of *S. oualaniensis*, largely avoided the primer mismatch with traditional Folmer primers.

Universal 16S primers provide a simple test for DNA quality because they amplify a product that is similar in size to the COI gene. As a result, screening samples that fail to amplify COI with 16S provide a quick check for amplifiable DNA. When both COI and 16S fail, this likely reflects DNA degradation or the presence of PCR inhibitors. In our case, 36 PCR bands were amplified with the 16S primer set for all specimens indicating that DNA templates were generally high quality (Figure 1), but DNA from four ‘dwarf form’ specimens failed to amplify 16S, suggesting DNA degradation. Nevertheless, strong PCR products were generated with our new COI primer set for all specimens from two morphologically distinct forms (phenogroups) of *S. oualaniensis* and exhibited clear sequencing signal, even the template from four ‘dwarf form’ specimens failed to amplify 16S. Our result suggested the new *Sthenoteuthis* – specific primer set enable both efficient polymerase chain reaction amplification of the COI barcode region and that deliver high-quality sequence data.

In summary, the primer set developed in this study is highly effective in generating amplicons that sequence cleanly for the COI barcode region of two morphological groups of *S. oualaniensis*. The new primer set will facilitate further studies in phylogeny and cryptic diversity of *S. oualaniensis.*
